# Re-Sharpening Files

**Published:** 1881-07

**Authors:** M. Werdermrnn


### RE-SHARPENING FILES.

Clean carefully with hot water and soda; then place in connection with the positive pole of a battery, in a bath composed of

| | | |
|---|---|---|
| Sulphuric acid...... | 40 | parts |
| Nitric acid.......... | 80 | " |
| Water........... | 1,000 | " |

The negative pole is formed of a copper spiral surrounding the files, but not touching them; the coil terminates in a wire which rises toward the surface. This arrangement is the result of practical experience. When the files have been ten minutes in the bath they are taken out, washed and dried, when the whole of the hollows will be found to have been attacked very sensibly; but should the effect not be sufficient, they are replaced for the same period as before. M. WERDERMRNN.

